# Triage of Patients Consulted for ICU Admission During Times of ICU-Bed Shortage

**DOI:** 10.14740/jocmr1939w

**Published:** 2014-09-09

**Authors:** Jose Orsini, Christa Blaak, Angela Yeh, Xavier Fonseca, Tanya Helm, Ashvin Butala, Joaquin Morante

**Affiliations:** a Department of Medicine, New York University School of Medicine, Woodhull Medical and Mental Health Center, 760 Broadway, Brooklyn, NY 11206, USA

**Keywords:** Triage, Intensive care unit, Step-down unit, Emergency department, Acute physiology and chronic health evaluation, ICU beds

## Abstract

**Background:**

The demand for specialized medical services such as critical care often exceeds availability, thus rationing of intensive care unit (ICU) beds commonly leads to difficult triage decisions. Many factors can play a role in the decision to admit a patient to the ICU, including severity of illness and the need for specific treatments limited to these units. Although triage decisions would be based solely on patient and institutional level factors, it is likely that intensivists make different decisions when there are fewer ICU beds available. The objective of this study is to evaluate the characteristics of patients referred for ICU admission during times of limited beds availability.

**Methods:**

A single center, prospective, observational study was conducted among consecutive patients in whom an evaluation for ICU admission was requested during times of ICU overcrowding, which comprised the months of April and May 2014.

**Results:**

A total of 95 patients were evaluated for possible ICU admission during the study period. Their mean APACHE-II score was 16.8 (median 16, range 3 - 36). Sixty-four patients (67.4%) were accepted to ICU, 18 patients (18.9%) were triaged to SDU, and 13 patients (13.7%) were admitted to hospital wards. ICU had no beds available 24 times (39.3%) during the study period, and in 39 opportunities (63.9%) only one bed was available. Twenty-four patients (25.3%) were evaluated when there were no available beds, and eight of those patients (33%) were admitted to ICU. A total of 17 patients (17.9%) died in the hospital, and 15 (23.4%) expired in ICU.

**Conclusion:**

ICU beds are a scarce resource for which demand periodically exceeds supply, raising concerns about mechanisms for resource allocation during times of limited beds availability. At our institution, triage decisions were not related to the number of available beds in ICU, age, or gender. A linear correlation was observed between severity of illness, expressed by APACHE-II scores, and the likelihood of being admitted to ICU. Alternative locations outside the ICU in which care for critically ill patients could be delivered should be considered during times of extreme ICU-bed shortage.

## Introduction

Healthcare resource allocation refers to the distribution of healthcare resources and encompasses rationing and triage. With more complex medical procedures, increasing patient age and expectations, and the increased severity of diseases, there is a greater demand for all medical services. Given all these conditions, occasionally the need for close monitoring is required in some patients. When it comes to intensive care services, periodically demands exceed supply leading to a rationing of intensive care unit (ICU) beds. Although the tenets of biomedical ethics and international law indicate that protocols should be used to guide resource allocation when demand for ICU resources exceeds availability [1, 2], formal triage protocols are not routinely part of decision making surrounding patient ICU admission [3]. Decreased hospital beds availability and fiscal constraints are forcing ICUs to alter their approaches to triage. National and local organizations are mandating that hospitals comply with resource intensive and arguably unproven initiatives to monitor and improve patient safety and quality. Several studies have shown that decreased ICU beds availability is associated with a decreased likelihood of ICU admission [4-8]. When faced with ICU capacity constraints, hospitals have a limited number of options. One of them, and perhaps most obvious, is to add more ICU beds. However, this approach is misguided and potentially harmful, since increasing the number of ICU beds increases the hospitals’ fixed costs while creating waste in the system during times when ICUs are not full [9]. There are seven times as many adult ICU beds in USA than in United Kingdom, and approximately 20% of acute hospital admissions received intensive care in USA compared with only 2% in the United Kingdom [10, 11]. A large study showed that medical patients admitted to the ICUs in USA had lower severity of illness and were more likely to be admitted directly from an emergency department (ED), consistent with a picture where the threshold for admission in USA is lowered substantially when more ICU beds are available [12]. ICU beds availability varies widely worldwide, ranging from less than 1 to greater than 30 ICU beds per 100,000 people [13]. Despite this enormous variation, there is no consensus of the ideal number of ICU beds to serve a population. In theory, the number of ICU beds needed should be estimated based on the number of patients who were or should have been admitted to the ICU, the number of patients transferred to ICUs in other hospitals, and the ICU beds occupancy rates [14, 15]. In this report, the authors evaluated the characteristics of patients referred for admission to ICU during times of severely constringed beds availability.

## Methods

The study has a prospective, observational design. It was conducted in a general, community inner-city hospital located in Brooklyn, New York. The hospital has a total of 346 beds distributed among general internal medicine, general surgery, psychiatry, obstetrics/gynecology, and pediatrics wards. As per institutional policies, patients in whom mechanical ventilation and/or vasoactive therapy is required should be admitted either to ICU or step-down unit (SDU). The ICU in our hospital is a closed unit composed of 12 beds, with an annual admission average of 700 patients. The critical care medicine (CCM) service is constituted by five board-certified intensivists who provided 16 h a day in-house coverage that includes weekends and holidays. A senior medical resident evaluates all ICU consultations, and the CCM attending is required to assess every patient for whom a consultation is requested. The CCM team received notification and approved all the admissions to the ICU. In addition, the hospital has a 12-bed SDU which is supervised as well by the CCM team.

ICU overcrowding was defined as availability of two beds or less at any time during the study period. Available ICU beds were defined as the number of beds funded to be operational and without any assigned patient at the time the CCM evaluation was requested. Patients triaged to SDU and hospital wards were considered as refused from ICU admission.

All patients, 18 years or older, in whom an evaluation for ICU admission was requested during times of ICU overcrowding were included in the study. Triage decisions were based on the clinical determination by intensivists as well as the institutional requirements and criteria for admission to ICU. In patients readmitted to ICU within 30 days, only data from the first admission were analyzed. Each day, the number of available ICU beds and CCM requested evaluations were recorded. Variables such as age, gender, hospital location from where the consultation was requested, APACHE-II scores and diagnoses at triage, ICU beds available at the time of evaluation, triage disposition, and outcome at ICU and hospital discharge were measured. Electronic medical records were reviewed and clinical information was abstracted for each patient. After waiving the needs for informed consent, institutional review board approved the study.

## Results

A total of 95 patients were evaluated for ICU admission during the time of ICU overcrowding at our institution. Sixty-four patients (67.4%) were admitted to ICU, 18 (18.9%) were triaged to SDU, and 13 (13.7%) were admitted to hospital wards. Their mean age was 57.2 years (median 57, range 21 - 94), and majority were males (60, 63.2%). Two patients (3.1%) were readmitted to ICU within 30 days of their first admission. Locations from where ICU evaluations were requested were as follows: ED (57 patients, 60%), hospital wards (27 patients, 28.4%), OR/PACU (six patients, 6.3%), and SDU (five patients, 5.3%). Most common diagnoses at the time of triage are summarized in Table 1. Their mean APACHE-II score at the time of evaluation by CCM team was 16.8 (median 16, range 3 - 36). Mean APACHE-II score for patients admitted to ICU was 18.6 (median 18, range 3 - 36), their mean age was 58.7 years (median 61, range 24 - 94), and majority were males (40, 62.5%). Mean APACHE-II score for patients who were refused for ICU admission was 13.7 (median 14, range 3 - 28), their mean age was 54.1 years (median 54, range 21 - 83), and most were males (25, 80.6%). Mean APACHE-II score for patients who expired in ICU was 21.7 (median 21, range 7 - 36), their mean age was 59 years (median 64, range 24 - 94), and most were females (9, 60%). The total number of ICU beds available during the study period was 103: the ICU had no beds available in 24 opportunities (39.3%), one bed available in 39 (63.9%), and two beds available in 32 (52.5%). The mean APACHE-II score for patients evaluated when no beds were available was 17.4 (median 15, range 3 - 33), 16.9 (median 17, range 3 - 36) when only one bed was available, and 17.1 (median 15, range 3 - 33) when two beds were available. Triage disposition based on the number of available beds is shown in Table 2. Overall hospital mortality was 17.9%, while ICU mortality was 23.4%.

**Table 1:** Most Common Diagnoses at Triage

1) Respiratory failure: 26 patients (27.4%)
2) Shock: 22 patients (23.2%)
3) Cardiac arrest: eight patients (8.4%)
4) Electrolyte imbalance (diabetic ketoacidosis): six patients (6.3%)
5) Stroke: five patients (5.3%)
6) Gastrointestinal hemorrhage: five patients (5.3%)
7) Delirium tremens: five patients (5.3%)
8) Drug overdose: three patients (3.2%)

**Table 2:** Triage Dispositions Based on Number of ICU Beds Available

1) No ICU beds available
ICU: eight patients (33.3%)
SDU: nine patients (37.5%)
Hospital wards: seven patients (29.2%)
2) One ICU bed available
ICU: 27 patients (69.2%)
SDU: nine patients (23.1%)
Hospital wards: 3 patients (7.7%)
3) Two ICU beds available
ICU: 29 patients (90.6%)
Hospital wards: three patients (9.4%)
SDU: 0 patients (0%)

## Discussion

This study examined the characteristics of patients referred for ICU admission during times of limited beds availability. To our knowledge, this is the first report conducted in New York State related to triage of critically ill patients in circumstances where the number of ICU beds available was decreased. It is also one of the few studies in USA exploring the characteristics of patients evaluated for ICU admission under such circumstances.

ICU bed availability may affect triage, as bed allocation has been considered one of the most difficult and stressful aspects of ICU work [7, 16]. Although ICU triage decisions may have a major impact on patient outcomes and healthcare costs, few studies have focused on the outcomes of patients who are refused ICU admission [17, 18]. The percentage of patients refused ICU admission has ranged from 24% to 57%, bust most of the studies failed to distinguish among the reasons for ICU refusal [7, 8, 19-21]. Patients may be refused ICU admission because they are deemed too well or too sick to benefit from ICU management, the patient or family does not want ICU admission, the ICU is too busy to provide optimal care, or no ICU beds are available. In most studies, a full ICU explained only a small proportion of ICU refusals [7, 8, 20]. Previous reports have shown that, when fewer ICU beds are available, fewer patients are admitted and they are more severely ill [7, 17, 18, 22]. Our results failed to support these findings: 33% of patients evaluated for admission at times when the ICU had no available beds were admitted to ICU, while 69% of patients whom were assessed when only one bed was available were admitted to the unit. The mean APACHE-II scores for those patients was 21.8 and 19.5 (median 20 and 21, range 10 - 33 and 7 - 36), respectively. Moreover, 91% of patients evaluated for admission to ICU at times when two beds were available ended up been admitted to ICU. Their mean APACHE-II score was 16.9 (median 16, range 3 - 33). Having different clinical thresholds for patients’ ICU admission based on physician or health system factors may result in misallocation of patients to hospital units and may compromise the quality and efficiency of healthcare delivery. When there were no available beds, 38% of patients were triaged to SDU, and when only one bed was available 23% of patients were admitted to that unit. These results may be interpreted as that our triaging intensivists made safe practice decisions by using an alternative monitored setting when the ICU was overcrowded. Similar to majority of ICUs in USA, the CCM division at our institution has written criteria for admission. These criteria are based mainly on physiologic parameters (blood pressure, heart rate, respiratory rate), and the needs for mechanical ventilator support and/or vasoactive therapy. However, as has been published, majority of intensivists do not strictly follow these criteria to make daily ICU admission decisions. Rather, decisions as to whether to admit or not patients to ICU often rest on the judgment of the consulting CCM physician. Balancing patients’ needs for critical care with the available critical care resources is a great challenge. Triage strategies developed to assist in admission decisions may be particularly useful when the ICU is full. The strategies should comply with ethical principles to ensure that patients have equal access to ICU management regardless of their personal and behavioral characteristics. Mechanisms are required to increase the flexibility of critical care resources in systems that operate near capacity. Alternatives strategies could include creating temporary ICU resources by transferring or admitting patients to other locations in the hospital that are suitable for advanced cardiopulmonary resuscitation and monitoring. Although their cost-effectiveness has not been demonstrated, intermediate care units offer theoretical advantages for patients needing monitoring and less intense nursing care, and that would give intensivists flexibility during the triage process resulting in more appropriate use of ICU beds [23, 24]. PACUs may function clinically as an ICU during times of overcrowding, although these units were neither designed, staffed, nor equipped to serves as an ICU [25, 26]. We cannot explain why our ICU was overcrowded during those 2 months, although we hypothesized that local and geographic factors may have played a role.

When ICUs are strained, triage decisions seem to be affected such that patients are discharged or transferred from the ICU more quickly and, perhaps consequently, have slightly greater odds of being readmitted to the ICU. However, short-term patient outcomes are unaffected, suggesting that bed availability pressures may encourage intensivists to discharge patients from ICU more efficiently [27]. Our readmission rate of 3.1% falls into rates reported previously, which ranged from 0.9% to 19% [28, 29].

The unadjusted ICU mortality in our study is comparable to previously reported in one cohort [5]. The mean age for those ICU non-survivors was 59 years (median 64, range 21 - 94), and majority were females (9, 60%). Mean APACHE-II score for those patients was 21.7 (median 21, range 7 - 36). Our unadjusted hospital mortality is comparable to results reported by Wunsch et al [12], but it was significantly lower than previously published in one study [21].

This study has several limitations. First, it was conducted in a single institution and our findings may not be applicable to other ICUs with varying size and staffing models, since allocation of ICU resources and decision-making processes for patients’ goals of care may vary across healthcare jurisdictions. However, in this study we analyzed only the impact of ICU bed shortage under real-life conditions. Second, despite the large number of consecutive patients recruited, the study may have been underpowered and a larger sample size obtained by extending the study period would perhaps have revealed additional significant results. Third, there were no data collected on reasons for refusal of patients not admitted to ICU and neither was on long-term outcome after their hospital discharged. Fourth, we did not address the outcome of patients who were transferred from ICU to other locations in the hospital during times when beds were limited, in order to create beds for patients in needs. The main strength of this study is its prospective design, with evaluation of all consecutive patients in whom admission to ICU was requested during times of limited beds availability.

### Conclusion

ICU beds availability varies substantially, making physicians triage decisions difficult at times. The effect of ICU beds availability on patients outside the ICU is unknown. We must recognize that the relative provision of intensive care may be driven by many factors, including intensivists’ perceptions, heterogeneity in patients’ characteristics, and institutional policies. Alternatives hospital locations such as SDUs, intermediate care units, and PACUs might be acceptable for the delivery of critical care in an environment of constrained ICU bed access, and would allow CCM physicians a more rational distribution of resources. Further studies are warranted to evaluate the outcome of patients “bumped” to hospital wards in order to create ICU beds to accommodate new patients in needs of critical care.
